# Effectiveness of combined exercise intervention on sedentary behaviour patterns, body composition, and cardiometabolic health among middle-aged adults

**DOI:** 10.1186/s13102-025-01488-6

**Published:** 2025-12-28

**Authors:** Boyi Zhang, Ziteng Yang, Mingyue Yin, Yuting Xie, Hui Zuo, Tao Zhang, Siman Lei, Chuanzhi Wang, Sulin Cheng, Xiuqiang Wang

**Affiliations:** 1 Institute of Cardiology and Sports Medicine, German Sport University Cologne, Cologne, Germany; 2 Exercise Translational Medicine Center, National Center for Translational Medicine (Shanghai), Shanghai Jiao Tong University, Shanghai, China; 3 Montgomery High School, Montgomery, USA; 4 The Mary MacKillop Institute for Health Research, Australian Catholic University, Melbourne, Australia; 5 Faculty of Education, University of Macau, Macau SAR, China; 6 Lab of Regenerative Medicine in Sports Science, School of Physical Education and Sports Science, South China Normal University, Guangzhou, China; 7 Faculty of Sport and Health Sciences, University of Jyväskylä, Jyväskylä, Finland

**Keywords:** Sedentary behaviour, Accumulation patterns, Exercise intervention, Cardiometabolic, Physical activity

## Abstract

**Objectives:**

Sedentary behaviour (SB) presents a health risk independent of age, sex, and clinical disease status, while exercise interventions have been shown to potentially reduce sitting time. This study examined the effectiveness of a combined moderate-intensity continuous training (MICT) and high-intensity interval training (HIIT) program on SB patterns, body composition, and exercise performance among middle-aged adults.

**Design:**

Quasi-experimental intervention study without a control group.

**Methods:**

Thirty-two adults (50% women, aged 38.25 ± 8.27) completed a 7-week program with three weekly sessions of cycling (MICT) and rowing (HIIT). Baseline accelerometer data were used to classify participants into Breaker (≤ 15 min sedentary bouts), Intermediator (15–20 min), and Prolonger (≥ 20 min) groups. Paired-sample t-tests and repeated measures ANOVAs assessed changes, with effect sizes reported as η²p and Cohen’s d.

**Results:**

After intervention, the Breaker group showed increased breaks per sedentary hour and greater time in moderate-to-vigorous physical activity (MVPA) compared with Intermediator and Prolonger groups (all *p* < 0.05). Breakers also reduced body fat percentage, waist–hip ratio, and total cholesterol (*p* < 0.05–0.01), alongside improved maximum cycling (*p* = 0.001) and rowing load (*p* = 0.02). The Intermediator group demonstrated a reduction in body fat percentage (*p* = 0.005). However, mean sedentary bout duration, reflecting sedentary behaviour accumulation patterns, did not change significantly in any group (*p* > 0.05).

**Conclusions:**

The 7-week combined exercise training improved body composition, cardiometabolic profile, and exercise performance but did not alter sedentary behaviour patterns in middle-aged adults. Nonetheless, individuals with shorter sedentary bouts experienced significant health and performance benefits than those with prolonged bouts, suggesting that baseline sedentary profiles may moderate responsiveness to exercise interventions.

**Supplementary Information:**

The online version contains supplementary material available at 10.1186/s13102-025-01488-6.

## What is already known on this topic

Large cross-sectional studies have shown that different sedentary accumulation patterns lead to differences in cardiovascular metabolic capacity and physical fitness as well as all-cause mortality. The mean sedentary bout duration has been shown to be a valid parameter indicating sedentary pattern classification for predicting cardiovascular disease and cancer risk.

### What this study adds

The 7-week combined exercise intervention in a free-living setting is not sufficient to induce sedentary behavior change in middle-aged adults. However, people with short sedentary bout duration during intervention got significant health benefits and improved performance compared to those with prolonged sedentary bout duration counterparts.

## How this study might affect research, practice or policy

In sedentary behavior guidelines should take into account the intervention type and duration as well as sedentary behavior pattern to optimize people’s health benefits.

## Introduction

Sedentary behaviour (SB) is a significant contributor to increased risk of all-cause mortality and cardiovascular disease (CVD) in adults and elderly [1]. Current guidelines recommend that adults engage in more than 150 min of moderate to vigorous physical activity (MVPA) per week and limit total sedentary time to promote health, suggesting that replacing sitting with any type or intensity of physical activity (PA) is beneficial [2]. However, focusing only on PA and total SB time may be insufficient [3, 4]. Emerging evidence indicates that how sedentary time accumulates across the day, i.e., SB patterns also matters for health [5–8]. SB pattern refers to how sedentary time is accumulated over the day or waking week, encompassing factors such as timing, duration, frequency of sedentary bouts, and the number of breaks in sitting time. [9].

Observational studies have shown that, for a given total sedentary time, more frequent interruptions and shorter sedentary bouts are associated with more favourable cardiometabolic profiles, including lower waist circumference, body mass index, triglycerides, and improved lipid profiles, independent of MVPA [10, 11]. Mean sedentary bout duration has also been linked to long-term CVD risk [12]. These findings suggest that sedentary patterns and total sedentary time may have differential predictive value for chronic disease and all-cause mortality and that targeting patterns could be important for designing interventions to reduce SB. However, much of the current evidence is based on cross-sectional designs or laboratory-based protocols [13, 14], and some mechanistic work has been conducted in animals, [15] limiting causal inference and ecological validity.

The effectiveness of structured exercise program in increasing PA and reducing SB, particular in SB accumulation patterns, remains uncertain. In a 2-week intervention in overweight adults, high-intensity interval training (HIIT) and moderate-intensity continuous training (MICT) both produced modest reductions in total sitting time, but no meaningful changes in SB accumulation parameters such as mean sedentary bout duration were observed [16]. Another study randomly assigned young adults to intervention and control groups, and found no significant group differences in SB after 12 weeks of aerobic and muscle-strengthening training intervention [17]. Moreover, these studies typically examined MICT or HIIT in isolation. Previous research has suggested that combining HIIT and MICT may yield greater improvements in metabolic health and body composition among middle-aged adults than either modality alone [18], yet it is unclear whether such combined, individualized exercise programmes can modify SB accumulation patterns, or whether potential benefits differ by baseline sedentary profile.

Therefore, the present study aims to investigate the effects of a 7-week combined exercise intervention, incorporating both HIIT and MICT, on SB accumulation patterns, body composition and cardiometabolic health among middle-aged adults. By comparing outcomes across groups with different sedentary accumulation profiles, this study seeks to evaluate the potential of a real-world, structured, combined-exercise program to promote meaningful behavioral and physiological improvements.

## Methods

### Study design and participants

This 7-week intervention study, with a quasi-experimental design, was part of a clinical trial project titled “Application of Exercise Intervention and Health Management in Sedentary Population” (registration number: ChiCTR2100047173). This study was approved by the Ethic Committee of Shanghai Jiao Tong University (reference number: B2020025I). Informed consent was obtained from all participants prior from the laboratory tests and data collections. This study was reported in accordance with the CONSORT guidelines [19]. A completed CONSORT checklist is provided as an additional file (Supplementary file 2).

Participants were recruited at Shanghai Jiao Tong University through offline presentations, posters, and the social media platform WeChat. Participants were eligible for this study if they were aged between 30 and 65, spent most of their day sitting (confirmed by a standardized interview), were free from other medical conditions, and had not engaged in regular exercise for the past three months (less than two sessions per week, each lasting less than 30 min). We excluded those who had a body mass index (BMI) above 40 kg/m^2^, suffered from severe cardiovascular disease, skeletal muscle disease, or psychological problems, were diagnosed with Type I or Type II diabetes and had other prohibitions to exercise.

**Fig. 1:**
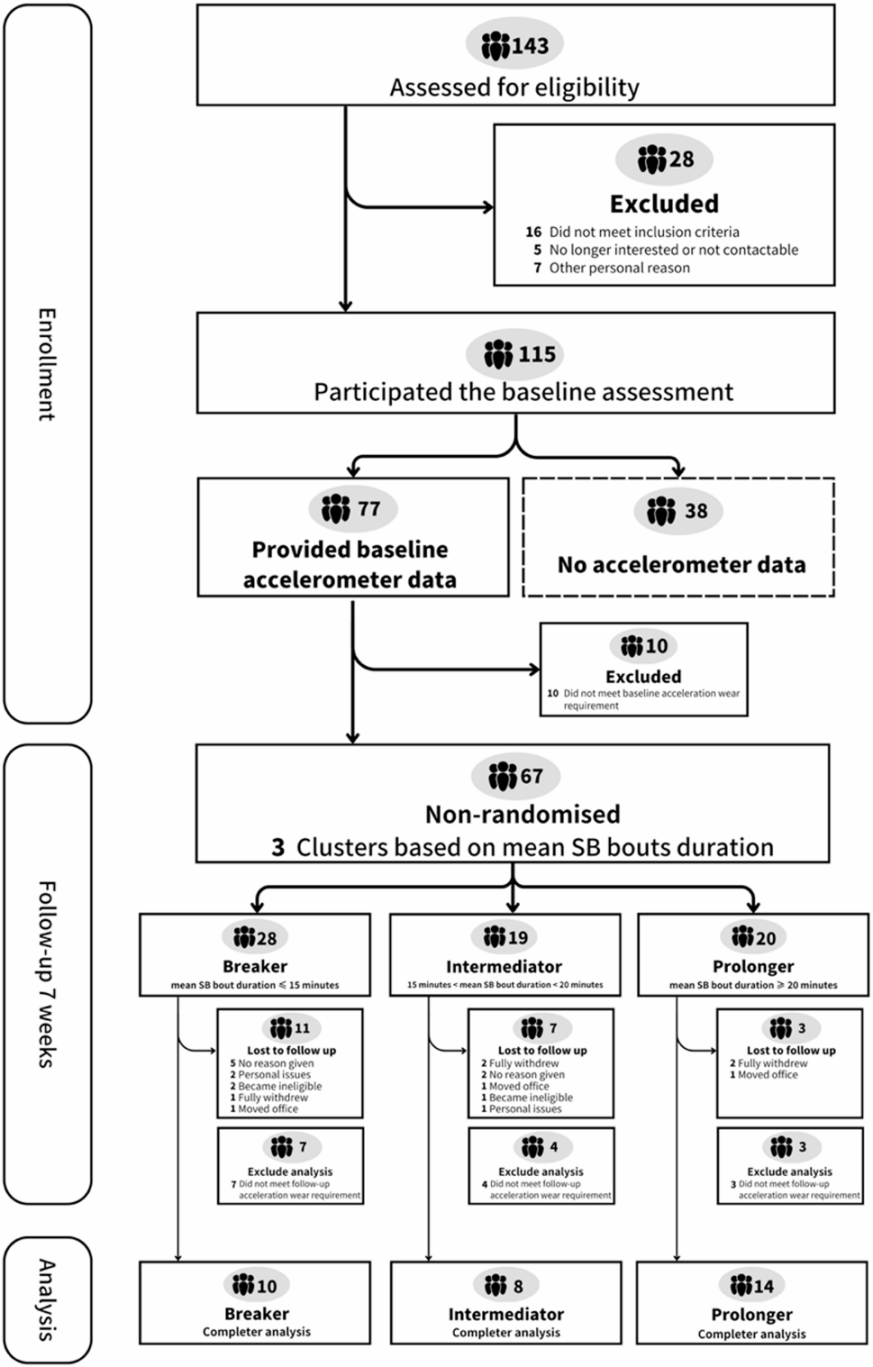
Flowchart of study design and participants’ recruitment

From December 2020 to May 2021, 143 participants were invited to participate in this study to objectively measure their SB patterns and complete questionnaires regarding their demographic profiles and other self-reported outcomes. Out of the 143 participants, a total of 115 participants agreed to participate in the study, and 77 participants were further agreed to wear an ActiGraph GT9X Link accelerometer (ActiGraph, Pensacola, FL), which was provided to be worn on their non-dominant hip. The accelerometer was worn continuously for seven consecutive days before and after they finish the exercise intervention, except sleeping or during water activities such as swimming, showering. No adverse events occurred during the intervention in this study. Detailed information on study design and participant recruitment can be found in Fig. 1.

### Accelerometer data processing

Raw triaxial accelerometer data (recorded at 30 Hz in 60-second epochs, with a dynamic range of ± 8 g) were processed in ActiLife software (Actigraph, Pensacola, FL, Version 6.13.4). Non-wear periods were defined as consecutive 0 counts lasting at least 90 min, allowing for a drop time window (≤ 2 min) if an entire absence of movement was observed during the first 30 min and the following 30 min [20]. Valid data are considered as wearing the device for at least 3 days on weekdays and at least 1 day on weekends (total number of days = 4) and at least 10 h per day [21]. The mean valid accelerometer days were 6.66 (SD: 0.94) at baseline and 6.75 (SD: 0.67) at posttest. Total valid minutes during the recording days were 909.04 (SD: 77.35) in the baseline period and 906.14 (SD: 97.05) in the posttest phase.

### Free-living sedentary time and physical activity intensities

After validated the wearing days, SB was identified as all valid wearing time spent ≤ 99 counts per minute (CPM) [22], and bouts were defined as periods of consecutive sedentary lasting ≥ 5 min [12]. The total sedentary time was calculated by adding up the minutes during which the accelerometer was worn, and the participant was sedentary. A break was defined as any interruption in sedentary time lasting at least 1 min and causing the accelerometer count to reach or exceed 99 counts per minute. PA intensities, including light physical activity (LPA) and moderate to vigorous physical activity (MVPA), were determined by employing previously established and validated cutpoints: LPA (100–2019 CPM) and MVPA (≥ 2020 CPM) [22].

### Sedentary behaviour accumulation patterns and breaks

The percentage of SB time (SB %) was obtained by dividing total sedentary time by daily valid wear time and multiplying by 100. Breaks per sedentary hour, also known as the fragmentation index or break rate, were calculated by dividing the number of sedentary breaks by total sedentary time in hours [23]. Mean sedentary bouts duration was derived from the number of sets of SB (n sedentary bouts) over 5 min and the total duration of SB, based on the following formula developed from recent work: $\:\frac{\sum\:time\:in\:SB\:Bouts\:}{n\:SB\:bouts}$ [12]. Higher values of this metric indicate less fragmentation of sedentary time [5]. According to the previous studies that highlighted the relevance of bout duration for cardiometabolic risk and proposed similar cut-off values [12], we classified participants into three groups according to their mean sedentary bout duration. Participants with a mean sedentary bout duration of 15 min or less were classified as Breakers, those with 15 to less than 20 min as Intermediators, and those with 20 min or more as Prolongers. This approach was chosen to provide conceptually meaningful categories while maintaining sufficient group sizes for analysis.

### Cardiometabolic health and body composition

#### Anthropology body composition

Height was assessed using a wall-mounted height-measuring tape and body weight and body composition outcomes were measured using bioelectrical impedance analysis (InBody 720, Korea). Measurements were conducted after an overnight fast in the morning using standard processure. Body fat percentage, waist-hip ratio (WHR), and skeletal muscle mass were extracted from the analyzer. The BMI was computed by dividing the body mass by the square of the body height (kg/m^2^).

### Lipid profiles

A fasting venous blood sample of 20 mL was collected from the anterior elbow vein in the morning (7–8 a.m.) after overnight fast. The serum was separated within 30 min and subsequently stored at −80 °C for further analysis. Total cholesterol (TC) and high-density lipoprotein levels (HDL) were assessed using an automated biochemistry analyzer (AU680, Beckman Coulter, USA). The total cholesterol to HDL ratio (TC/HDL ratio) was calculated [24].

### Blood pressure

Blood pressure and resting heart rate were measured twice at baseline and 7 weeks using an arterial stiffness testing device (bp-203rpe, Omron, Japan) in the supine position. After resting for 5 min, two measurements of systolic and diastolic blood pressure were taken in the supine position, and the mean value was used as the final result, reported in millimeters of mercury (mmHg).

### Exercise performance

The participants’ maximum training load (ML) was measured at baseline (week 0) to develop individualized exercise programs and at the end of the intervention (week 7). The assessments included cycling and rowing ML tests performed on the Monark LC7TT cycle ergometer (Monark, Sweden) and the DOMYOS 500 magnetic resistance rowing machine (Decathlon, France) on two different days.

The cycling test started with an initial exercise intensity of 50 W for male and 30 W for female lasting 3 min, with increments of 20 W in load every 3 min. Participants were required to maintain a pedal cadence of ≥ 60 RPM throughout the test until exhaustion or inability to sustain 60 RPM. In the magnetic resistance rowing machine test, the initial load intensity was 1 kg, with increments of 1 kg every 2 min. The rowing frequency was set at 26 times/minute and sustained until the participant reached exhaustion or was unable to maintain the 26 times/minute. In these two tests, heart rates were recorded with a Polar H10 heart rate belt (Polar Electro, Finland). The perceived exertion (RPE) rating was asked and evaluated using the Borg Subjective Fatigue Scale (6–20) after each exercise load and after the test.

### Combined exercise protocol

**Fig. 2:**
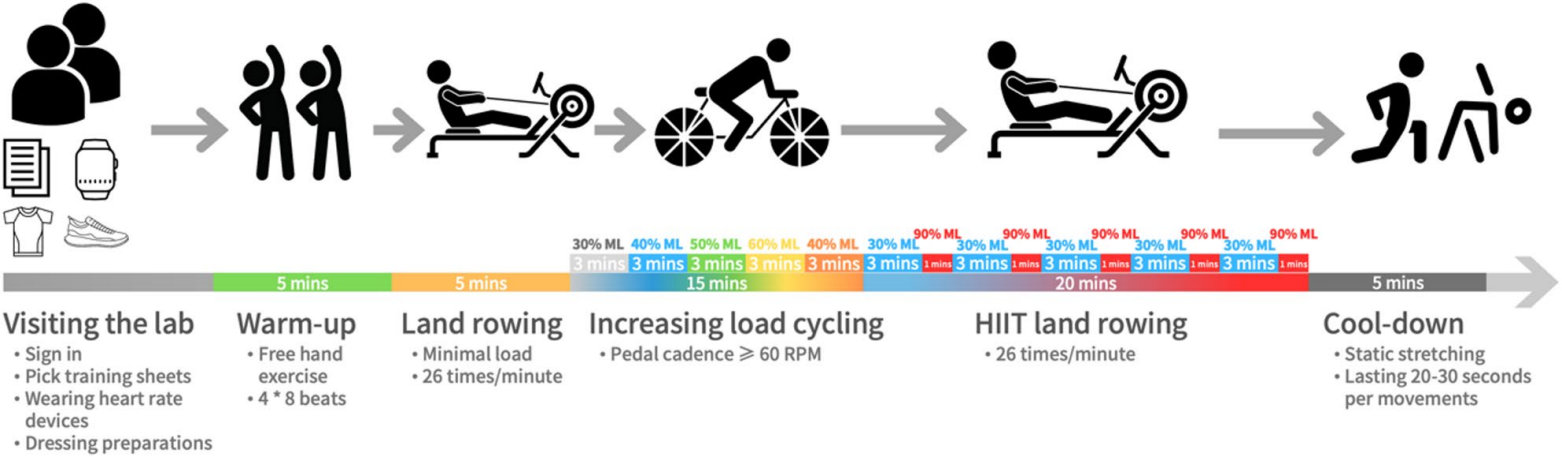
Exercise intervention procedure

During the 7-week intervention, participants completed three exercise sessions per week, with rest days scheduled between sessions to ensure adequate recovery (Fig. 2). Participants selected training days based on their personal availability, with most choosing to exercise during breaks in the workday. As a result, they typically trained in informal, randomly formed groups. All training sessions were conducted in a conveniently located indoor gym within the university office building and were supervised by a research assistant with a background in sports science. The combined exercise intervention in each session consisted of, a 10-minute warm-up completed by a 5-minute dynamic free-hand exercise and 5 min on a rowing machine. Followed by gym-based cycle with an increasing load of 40%, 50%, 60%, 65%, and 70% of the ML, respectively. Each exercise intensity lasted 3 min. After the cycling, participants engaged in five sets of HIIT, with each set consisting of 3 min of low intensity (40% of ML) and 1 min of high intensity (90% of ML) on a DOMYOS 500 magnetic resistance rowing machine (Decathlon, France) for 20 min. The rowing frequency was set at 26 times/minute. After the exercise, a 5-minute static stretch was performed in a standing position (Details of the training datasheet are available in Supplementary file 1).

### Monitoring training and engagement rates

During the intervention, the mean heart rate recorded in the training sessions was 124.7 ± 12.2 bpm (~ 68%HR_peak_), and the mean RPE was 14.3 ± 1.5 AU. No significant differences were observed in the internal training load between the groups (all *p* > 0.05). Before each training session, participants were required to sign in. No adherence-promoting strategies were implemented. Sign-in records were used to evaluate participant engagement, which was calculated using the following formula:

$$\:\frac{\mathrm{N}\mathrm{u}\mathrm{m}\mathrm{b}\mathrm{e}\mathrm{r}\:\mathrm{o}\mathrm{f}\:\mathrm{S}\mathrm{i}\mathrm{g}\mathrm{n}-\mathrm{i}\mathrm{n}\mathrm{s}}{21}\times100$$

### Statistical analysis

This self-controlled study has a large effect based on the G*Power sample size software (Version 3.1, University of Dusseldorf, Germany). For the setting of repeated-measures analysis of variances (ANOVAs, within-between interaction), effect sizes f = 0.4; a = 0.05; statistical power = 0.80, 3 groups, 2 measurements, correlation among repeated measurements 0.5. The required sample size to detect a change in mean sedentary bout duration was 7 in each group, which was met in this study.

Mean and standard deviation (SD) are reported for continuous variables, while numbers (n) and percentages (%) are presented for categorical variables. The final analytical sample was first defined using the primary accelerometer dataset by identifying participants with valid wear time at both baseline and post-intervention. All analyses were then performed using a complete-case, pairwise exclusion approach: for each outcome, only participants with data at both time points for that specific variable were included, and missing values were not imputed, so that each parameter was analysed on complete data. The Shapiro-Wilk test and visual inspection of Q–Q plots and residuals were conducted to determine the normality of the distribution. Baseline differences among the three sedentary accumulation pattern groups were examined using one-way ANOVA for continuous variables. To evaluate changes over time and group differences in SB profiles, a mixed repeated-measures 2 × 3 ANOVA was performed with time (pre, post) as the within-subject factor and sedentary profile group (Breaker, Intermediator, Prolonger) as the between-subject factor. Age, BMI, sex and engagement rate were included as covariates. Main effects and time-by-group interactions were examined. Levene’s test was used to assess homogeneity of variance. Bonferroni correction was applied to adjust for multiple comparisons. Effect sizes for ANOVA were expressed as partial eta-squared (η²p) and interpreted as small (0.01), medium (0.06) or large (0.14) [25]. Paired sample t-test was used to examine within-group intervention effects on variables related to body composition, lipid profiles, blood pressure, exercise performance and PA. Mean differences (MD) were computed to measure the magnitude of change over time. Cohen’s d was used to quantify effect size [26]. Statistical significance was considered at *p* < 0.05. All analyzes were performed using the R program (4.1 version, The R Project for Statistical Computing).

## Results

### Participants baseline characteristics

Table 1 presents the baseline characteristics of the participants. No significant differences among the three groups were observed in demographic or anthropometric variables (all *p* > 0.05).

**Table 1:** Sociodemographic characteristics of participants at baseline by SB patterns

	Total (*n* = 32)	Breaker (*n* = 10)	Intermediator (*n* = 8)	Prolonger (*n* = 14)
**Age (years)**	38.25 ± 8.27	39.0 ± 9.12	40.38 ± 11.1	36.5 ± 5.71
**Height (cm)**	166.84 ± 9.6	170.8 ± 8.75	165.13 ± 9.73	165 ± 9.90
**Weight (kg)**	74.66 ± 14.87	74.83 ± 10.72	77.24 ± 19.82	73.06 ± 15.15
**BMI (kg·m⁻²)**	26.33 ± 3.25	25.56 ± 2.65	26.84 ± 4.11	26.6 ± 3.25
**Women**,** n (%)**	16 (50.0)	3 (30.0)	4 (50.0)	9 (64.29)
**Education**,** n (%)**				
Bachelor’s degree	7 (21.88)	2 (28.57)	3 (42.86)	2 (28.57)
Master’s degree	11 (34.38)	2 (18.18)	3 (27.27)	6 (54.55)
PhD degree	14 (43.75)	6 (42.86)	2 (14.29)	6 (42.86)
**Smoking status**,** n (%)**				
Current	1 (3.13)	0	0	1 (100)
Former	2 (6.25)	1 (50)	1 (50)	0
Never	29 (90.63)	9 (31.03)	7 (24.14)	13 (44.83)
**Alcohol use**,** n (%)**				
Current	4 (12.5)	1 (25)	2 (50)	1 (25)
Former	1 (3.13)	1 (100)	0	0
Never	27 (84.38)	8 (29.63)	6 (22.22)	13 (48.15)

All continuous variable values are mean ± SD. All categorical variable values are n and %. BMI, body mass index

### Intervention effects on changes in free-living SB and PA profiles among sedentary patterns

**Fig. 3:**
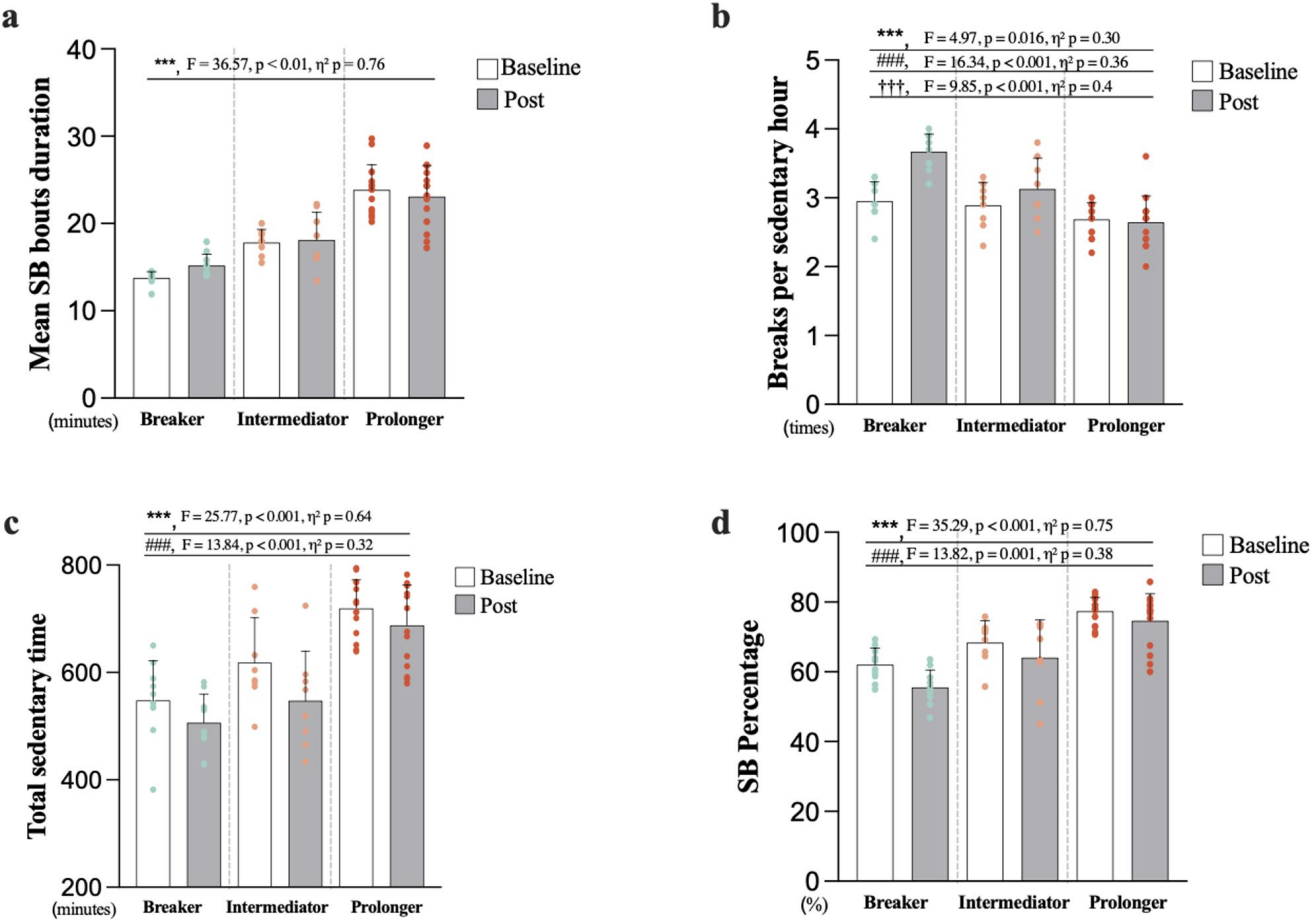
Changes in sedentary behaviour pattern profiles by groups. **a**–**d** including **a** mean sedentary bout duration, **b** breaks per sedentary hour, **c** total sedentary time, and **d** SB percentage at baseline and post-intervention. Data are presented as mean ± SD, with coloured dots indicating individual participants in each group.* indicates between-group differences from Bonferroni-adjusted post hoc comparisons (main effect of group): *, *p* < .05; **, *p* < .01; ***, *p* < .001. # indicates significant change over time (main effect of time): #, *p* < .05; ##, *p* < .01; ###, *p* < .001. † indicate significant time x group interaction effects: †, *p* < .05; ††, *p* < .01; †††, *p* < .001

Engagement in the 7-week combined exercise intervention did not differ significantly among the three groups (Breaker: 60.5 ± 22.3%; Intermediator: 68.2 ± 27.2%; Prolonger: 55.3 ± 26.6%; *p* > 0.05). For mean sedentary bout duration, no significant time-by-group interaction or main effect of time was observed (both *p* > 0.05; Fig. 3). However, there was large main effect of group on mean sedentary bout duration (F (2, 23) = 36.57, *p* < 0.01, η² *p* = 0.76), with Bonferroni post hoc tests confirming the expected ordering of groups (Breaker < Intermediator < Prolonger).

For other indicators of SB profiles, we found a significant difference in the rate of breaks time (F = 16.34, *p* < 0.001, η² *p* = 0.36). The Breaker group exhibited a significantly higher break rate post-intervention (F = 9.85, *p* < 0.001, η² *p* = 0.4).

Total sedentary time significantly differed between groups (F = 25.77, *p* < 0.001, η² *p* = 0.64) and decreased from baseline to post-intervention (F = 13.84, *p* < 0.001, η² *p* = 0.32). The Prolonger group had a longer daily sedentary time compared to the Breaker (719.37 vs. 547.81 min, *p* < 0.001), whereas all groups showed reductions in total sedentary time and SB% over the intervention period. These changes are summarized in Fig. 3.

**Fig. 4:**
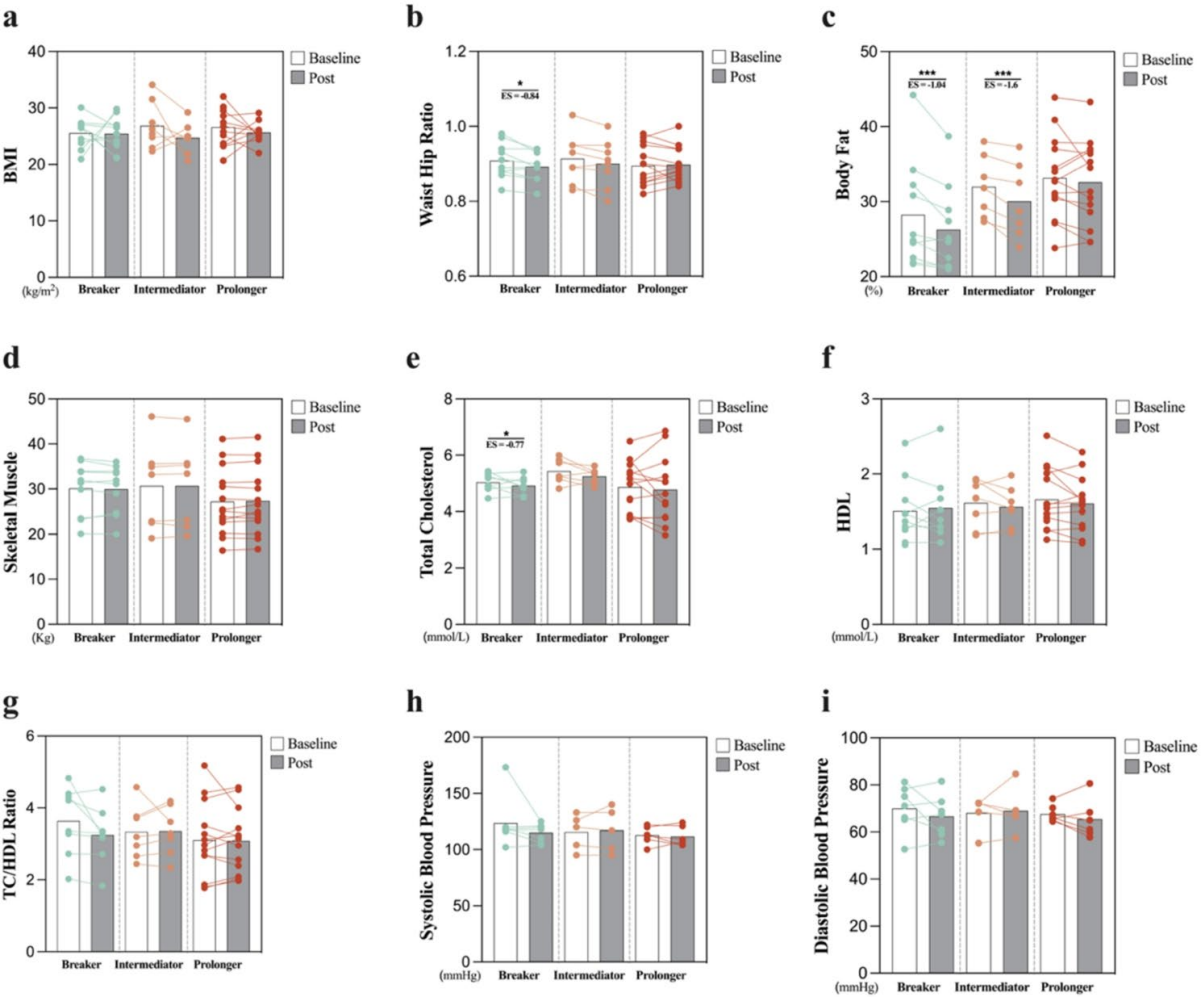
Intervention effects on body composition and lipid profiles among sedentary pattern groups.** a-d** The anthropometric and body composition changes. **e-g** The blood lipid profiles change. **h-i** The blood pressure profiles. Abbreviations: BMI, body mass index; TC, total cholesterol; HDL, high-density lipoprotein. *, *p* < 0.05; ***, *p* < 0.001

After the intervention, body fat percentage decreased significantly in the Breaker (MD = −1.97%, *p* < 0.01) and the Intermediator (MD = −1.93%, *p* < 0.01) groups. WHR decreased in the Breaker group (MD = −0.02, *p* = 0.026). However, no significant changes in body composition were found in the Prolonger group (all *p* > 0.05; Fig. 4). For blood lipids, TC decreased in the Breaker group (MD = −0.58 mmol/L, *p* = 0.049), while other blood lipid profiles and blood pressure did not change significantly in any group (all *p* > 0.05).

### Intervention effects on exercise performance and physical activity intensities

There was a significant increase in the maximal cycling load in both the Breaker (MD = 30 W, *p* < 0.001) and the Intermediator groups (MD = 7.5 W, *p* = 0.048), whereas only the Breaker group showed an improvement in maximal rowing load (MD 1.9 kg, *p* < 0.05).

In the PA-related variables, the Breaker group significantly increased their time spent in MVPA compared to baseline (MD = 37.25 min, *p* < 0.001), while no significant changes were observed in the Intermediator and Prolonger groups. Although there was a trend towards increased time in LPA, these changes were not statistically significant (all *p* > 0.05; Table 2).

**Table 2:** Changes in body composition, blood lipids, exercise performance and physical activity intensities

	Breaker		Intermediator				Prolonger		
Variables	MD (95% CI)	*p*	ES	MD (95% CI)	*p*	ES	MD (95% CI)	*p*	ES
BMI (kg·m⁻²)	-0.12 (-3.55 to 3.3)	0.938	0.03	-2.37 (-7.01 to 2.27)	0.258	0.47	-0.38 (-2.80 to 2.04)	0.734	0.11
Waist Hip Ratio	-0.02 (-0.03 to 0.00)	0.026*	0.84	-0.02 (-0.03 to 0.00)	0.052	0.91	0.00 (-0.01 to 0.02)	0.682	0.11
Body Fat %	-1.97 (-3.320 to -0.62)	0.009**	1.04	-1.93 (-3.04 to -0.82)	0.005**	1.6	-0.58 (-1.99 to 0.83)	0.39	0.24
Skeletal Muscle (kg)	-0.15 (-0.76 to 0.46)	0.594	0.17	0.00 (-0.50 to 0.50)	1	0	0.16 (-0.21 to 0.53)	0.377	0.24
Total Cholesterol (mmol·L⁻¹)	-0.58 (-1.15 to 0.00)	0.049*	0.77	-0.01 (-0.65 to 0.62)	0.958	0.02	-0.13 (-0.52 to 0.27)	0.5	0.19
TC/HDL Ratio	-0.36 (-0.8 to 0.08)	0.099	0.62	0.02 (-0.42 to 0.46)	0.915	0.04	-0.02 (-0.28 to 0.24)	0.874	0.05
HDL (mmol·L⁻¹)	-0.01 (-0.16 to 0.14)	0.878	0.05	-0.04 (-0.18 to 0.10)	0.479	0.29	-0.08 (-0.18 to 0.02)	0.113	0.47
Diastolic BP (mmHg)	-1.83 (-9.86 to 6.19)	0.583	0.24	0.92 (-7.84 to 9.68)	0.785	0.13	-1.75 (-7.31 to 3.81)	0.455	0.33
Systolic BP (mmHg)	-2.17 (-8.16 to 3.82)	0.395	0.38	1.60 (-7.21 to 10.41)	0.64	0.23	-1.17 (-7.34 to 5.00)	0.647	0.2
ML in rowing (kg)	1.9 ± 0.64 (0.45 to 3.35)	0.016*	0.94	1.0 ± 0.57 (-0.34 to 2.34)	0.121	0.62	0.21 ± 0.15 (-0.12 to 0.55)	0.189	0.37
ML in cycling (watt)	30 ± 6.32 (15.69 to 44.31)	0.001**	1.5	7.5 ± 3.13 (0.09 to 14.91)	0.048*	0.85	6.43 ± 3.41 (-0.95 to 13.8)	0.082	0.5
LPA (mins/day)	35.31 ± 21.28 (-12.83 to 83.45)	0.131	0.52	18.24 ± 17.69 (-23.6 to 60.07)	0.337	0.36	16.03 ± 16.53 (-19.67 to 51.74)	0.35	0.26
MVPA (mins/day)	37.25 ± 7.19 (20.98 to 53.53)	< 0.001***	1.64	8.87 ± 9.02 (-12.46 to 30.19)	0.358	0.35	8.59 ± 6.59 (-5.64 to 22.83)	0.215	0.35

Abbreviation: *BMI* Body mass index, *HDL* High-density lipoprotein, *TC* Total cholesterol, *BP* Blood pressure, *ML* Maximum load, *LPA* Light physical activity, *MVPA* Moderate to vigorous physical activity, *MD* Mean differences, *95%CI* Confidence interval at 95%, *ES* Effect size, refers to Cohen’s d; *, *p* < 0.05; **, *p* < 0.01; ***, *p* < 0.001

## Discussion

This study first compared changes in SB profiles, body composition-related health markers, and exercise performance across groups with different objectively measured sedentary patterns. Our main findings indicated that the 7-week combined MICT and HIIT intervention improved body composition, cardiometabolic markers and exercise performance, also reduced sedentary volume, but had minimal impact on SB accumulation patterns. Participants with shorter sedentary bouts at baseline tended to show more favourable adaptations than those with more prolonged bouts. Previous studies primarily focus on cross-sectional designs [5, 10, 12, 27, 28], with relatively few intervention studies and inconsistent results. Some trials have reported no significant changes in the indicators of SB after technology-based behavioural interventions, with SB accumulation patterns remaining largely stable in both intervention and control groups [29]. This is in line with our observation that mean sedentary bouts duration did not change significantly, despite reductions in sedentary volume (total sedentary time and SB percentage). In our study, the intervention was delivered in a gym located close to the workplace and could be performed around work breaks, which likely facilitated exercise participation. However, the stability of sedentary patterns suggests that simply offering exercise opportunities, even in convenient settings, may not be sufficient to modify how sedentary time is accumulated across the day. One alternative explanation relates to the compositional nature of time-ues data: increasing time in structured exercise will necessarily displace other behaviours within the 24-hour day and primarily reduce total sitting time or reallocate time to other activities or sleep, without changing the underlying pattern of sitting and breaks [30].

The stability of sedentary patterns may also be influenced by occupational demands [31]. Office-based employees typically spend prolonged periods seated and have a high prevalence of SB-related health risks [32]. Even when they participate in exercise programmes, they may compensate by reducing incidental movement or maintaining long uninterrupted sitting periods during working hours [33]. Previous research has suggested that structured physical activity may sometimes be accompanied by compensatory increases in sedentary time or reductions in light-intensity activity. This compensatory mechanism could partly explain why sedentary behaviour accumulation patterns remained unchanged in our sample, despite improvements in other outcomes [16]. Future studies should examine interventions that directly target sitting habits in the workplace, such as scheduled movement breaks or brief exercise snacks [34].

In terms of physiological outcomes, the 7-week combined exercise programme led to reductions in body fat percentage and waist-to-hip ratio, particularly in groups with higher participation in MVPA. The greater improvements observed in participants with shorter sedentary bouts (Breakers) are consistent with the notion that higher baseline movement fragmentation may support more favourable adaptations to training. The inclusion of HIIT-based rowing in the intervention likely contributed to improvements in body composition and cardiometabolic health, as previous work have shown that HIIT can enhance markers such as BMI, body fat percentage, blood pressure and resting heart rate [35, 36]. However, it should be noted that our HIIT modality (land-based rowing) and our sample (sedentary university staff) differ from those in many previous studies that used walking, running or cycling in more heterogeneous populations. These factors should be considered when comparing the magnitude of cardiometabolic benefits across studies. For blood lipids, we observed a reduction in TC in the Breaker group. Earlier work has indicated that increased sitting time and decreased PA are associated with unfavourable changes in lipid profiles, whereas reducing sedentary volume and interrupting sitting may improve TC [37, 38]. Our findings extend this evidence by showing that a relatively short, 7-week exercise intervention in a free-living, office-based population can improve total cholesterol in those with more fragmented sedentary patterns, alongside reductions in total sedentary time and increases in breaks.

Exercise performance generally improved across the SB pattern groups, yet significant improvements in maximal cycling and rowing load were mainly observed in the Breaker group. This observation suggests that individuals who already interrupt their sedentary time more frequently may be better positioned to take advantage of an added exercise stimulus. Improvements in physical fitness in this group may reflect a greater overall exposure to moderate- to high-intensity activity, both through the intervention sessions and through more active daily routines. [39]. These findings align with previous studies reporting strong associations between MVPA and cardiorespiratory fitness and support the importance of both structured exercise and a more active daily pattern for enhancing performance [40].

Taken together, these findings have several practical implications for office-based, sedentary populations. First, a combined MICT and HIIT programme of moderate duration can improve body composition, total sedentary time and selected cardiometabolic markers, even when sedentary behaviour patterns remain stable. Second, baseline sedentary patterns appear to moderate training responsiveness, suggesting that tailoring interventions according to existing sedentary profiles may be useful. Third, the lack of change in sedentary behaviour accumulation patterns indicates that exercise programmes alone are unlikely to shift how sedentary time is distributed throughout the day.

The primary strengths of this study lie in the implementation of a structured exercise programme that combines MICT and HIIT in a real-world, free-living setting, and in the use of objective measures to assess both SB and PA. In addition, comparing changes in SB, cardiometabolic health, exercise performance and PA across objectively defined SB accumulation pattern groups provides a more nuanced view of how exercise interventions interact with existing sedentary profiles. However, several limitations should be considered when interpreting the findings. First, participants did not wear accelerometers continuously throughout the whole 7-week intervention due to compliance and equipment constraints, which may limit the interpretation of SB patterns over time. Second, accelerometer data were processed using 60-second epochs and breaks were defined as periods of at least 1 min above the sedentary threshold. This setting may underestimate very short interruptions in sitting and inflate mean sedentary bout duration. Third, this quasi-experimental study did not include a non-exercising control group, which restricts the ability to draw strong causal inferences about the effects of the intervention. Fourth, the final sample size was relatively small and subgroup sizes were unequal, which reduces statistical power. Lastly, we did not systematically monitor dietary intake during the intervention, and these unmeasured lifestyle factors may have contributed to changes in body composition and cardiometabolic markers. Future studies with larger, controlled samples and comprehensive assessment of diet are needed to confirm and extend these results.

## Conclusion

The 7-week combined MICT and HIIT intervention was not sufficient to change the SB accumulation patterns among middle-aged adults. However, individuals with a sedentary profile characterized by shorter bout durations showed significant greater improvements in health and performance than those with more prolonged sedentary bouts, suggesting that baseline sedentary patterns may moderate responsiveness to exercise training. These findings indicate that combining structured exercise with targeted behavioural strategies, such as regular active breaks during work to reduce prolonged sedentary time, may be important to maximising health benefits.

## Supplementary Information

Supplementary Material 1.

Supplementary Material 2.

## Data Availability

The original contributions presented in the study are included in the article/supplementary material. Further inquiries can be directed to the corresponding author.

## Declarations

### **Clinical trial number**

ChiCTR2100047173 was accessed on June 9, 2021, via the Clinical Trial Registry at http://www.chictr.org.cn/.
