# Supplementary material for: Effectiveness of combined exercise intervention on sedentary behaviour patterns, body composition, and cardiometabolic health among middle-aged adults
Source: BMC Sports Sci Med Rehabil. 2025 Dec 28;18:45. doi: 10.1186/s13102-025-01488-6 (PMC12859953; doi:10.1186/s13102-025-01488-6)
Supplement: Supplementary file 1 — Supplementary Material 1. [file 13102_2025_1488_MOESM1_ESM.docx]

**Date： year – month- day （Weekday）**

**Participant ID: W001**

***Maximum Heart Rate (bpm) :*  Heart Rate Monitor & Watch ID:：________**

***Physical Condition***

***Please indicate any physical discomfort or whether you are currently in your menstrual period (if applicable, please describe):：________________***

| **Start Time** | **Exercise Mode & Sequence** | **Duration** | **Training Content & Intensity** | | **Resistance Level** |
| --- | --- | --- | --- | --- | --- |
|  | **Open Heart Rate Monitor** | **Heart Rate（bpm）：** | | | |
|  | **Warm-Up (WU)** | **Freehand, 3 - 5 mins** | | |  |
|  | **WU-Light rowing on water-resistance rowing machine** | **5 mins** | **Minimal resistance (Stroke rate: 26 strokes/min)** | | **\** |
| **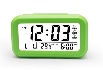** | **Cycle Ergometer（≥60RPM）**    **max load：190** | **3 mins** | **40% max load** | | **75 Watt** |
|  |  | **3 mins** | **50% max load** | | **95 Watt** |
|  |  | **3 mins** | **60% max load** | | **115 Watt** |
|  |  | **3 mins** | **65% max load** | | **125 Watt** |
|  |  | **3 mins** | **70% max load** | | **130 Watt** |
|  |  | **Immediate Post-Exercise Heart Rate（HR）** | | | **bpm** |
|  |  | **Immediate Post-Exercise Perceived Exertion（RPE）** | | |  |
| **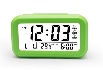** | **Magnetic resistance rowing machine**  **（Stroke rate: 26 strokes/min）**  **max load：11** | **3 mins** | **A** | **40% max load** | **4** |
|  |  | **1 mins** |  | **90% max load** | **10** |
|  |  | **3 mins** | **B** | **40% max load** | **4** |
|  |  | **1 mins** |  | **90% max load** | **10** |
|  |  | **3 mins** | **C** | **40% max load** | **4** |
|  |  | **1 mins** |  | **90% max load** | **10** |
|  |  | **3 mins** | **D** | **40% max load** | **4** |
|  |  | **1 mins** |  | **90% max load** | **10** |
|  |  | **3 mins** | **E** | **40% max load** | **4** |
|  |  | **1 mins** |  | **90% max load** | **10** |
|  |  | **Immediate Post-Exercise Heart Rate（HR）** | | | **bpm** |
|  |  | **Immediate Post-Exercise Perceived Exertion（RPE）** | | |  |
|  | **Cool Down and Stretching** | **2--3 mins** | | |  |
|  | **Close Heart Rate Monitor** | **Average Heart Rate（bpm）：** | | | |

**Notes：**

**(Research Assistant’s signature)**
